# Obituary: Ajit Dhaunchak, Ph.D

**DOI:** 10.3389/fncel.2013.00204

**Published:** 2013-11-08

**Authors:** Omar De Faria, Amit Bar-Or, Anshul Awasthi

**Affiliations:** Department of Neurology and Neurosurgery, Montreal Neurological Institute, McGill UniversityMontreal, QC, Canada

**Keywords:** Ajit Dhaunchak, myelin sheath, oligodendrocytes, montreal neurological institute, obituary


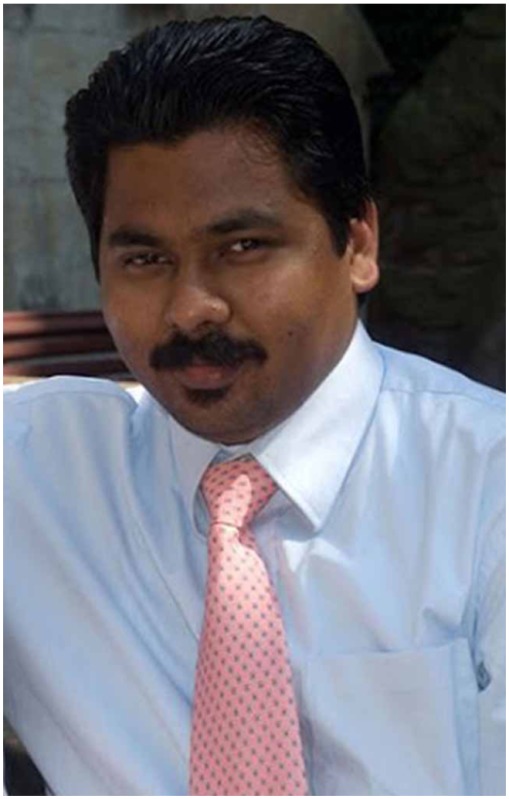


On the 18th of August 2012, Ajit Singh Dhaunchak passed away at the age of 33 in Quebec, Canada. His tragic and premature death was a terrible loss to his family, friends and colleagues, all of whom miss his dynamic personality and ever-smiling presence. At his funereal, Phil Barker, interim director of the Montreal Neurological Institute (MNI) at the time, emphasized how Ajit embodied that rare strain of scientist who combines gifted intellect, hard working, and seemingly endless stamina, with a most personable and charismatic personality—a remarkable set of qualities with which Ajit engaged and inspired all around him. Here, we wish to remember aspects of Ajit's professional life that reinforce Dr. Barker's observation and pay homage to a dear colleague and friend who, in all too short a life, already made important contributions to the field of neuroscience.

Ajit was born on August 1, 1979 in the city of Chandigarh, India, where he grew up and later entered the Panjab University to study Biophysics. Even as an undergraduate student he took a keen interest in understanding complex biological processes. When others were struggling to make sense of the course material, he could be seen reading books on protein chemistry and other topics well beyond the scope of the assigned course work. Apart from being an inspired student Ajit was also a gifted sportsman. Whether as an undergraduate student in India, a Ph.D student in Germany, or a postdoc in Canada—he always found time and friends to play his two favorite games—Cricket and Chess.

After graduating from Panjab University, Ajit left India, and moved to Germany where he enrolled in an integrated M.Sc-Ph.D program at the Max Planck Institute for Experimental Medicine. As a Ph.D student in the lab of Prof. Klaus-Armin Nave, he was introduced to the field of myelin biology and white matter diseases. His Ph.D research focused on the major myelin protein, proteolipid protein (PLP), and its mutations in association to Pelizaeus-Merzbacher disease (PMD). A paper published in *Journal of Cell Biology*, which summarizes part of the results obtained during his Ph.D, demonstrates that neuron-glia interactions regulate cell-surface expression of PLP in oligodendrocytes (Trajkovic et al., 2006). A second paper, published in *PNAS*, demonstrates that PMD-related mutations localized to the extracellular loop of PLP lead to protein cross-linking and PLP retention in the endoplasmic reticulum (Dhaunchak and Nave, 2007). Ajit was one of the very few students who managed to defend their Ph.D thesis in less than 3 years.

In 2007, soon after completing his Ph.D, Ajit joined the MNI and McGill University in Quebec, Canada, as a post-doctoral fellow in the laboratory of the late Dr. David R. Colman. There, Ajit continued to study PLP while also expanding his research interests and establishing several productive collaborations to investigate complementary aspects of white matter biology. He became particularly interested in the application of systems-biology approaches to the study of myelin and myelin diseases. In this context, Ajit made important contributions to our knowledge of the myelin proteome, lipidome, and transcriptome, both in health and disease. In work published in *Glia*, Ajit and colleagues provide a catalogue of novel proteins identified in axoglial specializations isolated from the human Central Nervous System (Dhaunchak et al., 2010). In another proteomic study lead by Ajit and featured in Annals of Neurology, a collaborative study by the Canadian Pediatric Demyelinating Disease Network provided evidence indicating that the myelin axoglial apparatus is disrupted at early stages of childhood-onset Multiple Sclerosis (Dhaunchak et al., 2012). In all, Ajit published eight papers during his post-doc in leading neuroscience journals and initiated or became involved in several other projects that continue even after his death.

During his last few months at the MNI, Ajit was busy writing project proposals and preparing for job interviews. Anyone listening to him practice a job talk had the distinct impression that in fact Ajit had already been running an active lab for several years, reflecting the large amount of quality data he had generated as well as his deep overall grasp of the field. There was no doubt Ajit was to become a most successful independent researcher, and everyone from fellow trainees to principle investigators at the MNI were certain that it's just a matter of time before he would get the position he wanted.

In addition to being respected by his peers as a most creative and dynamic researcher, Ajit was also recognized as a superb mentor for young(er) scientists in training. The most valuable proof of his mentoring skills is that in the last years of his career, David Colman trusted his Ph.D students to be trained by Ajit. As a mentor, Ajit not only provided the graduate students with the requisite detailed technical guidance and scientific training, but also infused in them his characteristic motivation, curiosity and drive for scientific excellence. Another indication of Ajit's talent was the steady stream of students and colleagues congregating at his desk for advice on their projects. A fair statement is that each and every laboratory in the MNI's Brain Tumor Research Centre (where the Colman lab was located) and multiple other labs at the Institute, benefited substantially from Ajit's generous and thoughtful input.

It was Ajit's ever-present smile and unfettered enthusiasm that captured the imagination, and indeed continues to inspire, his many friends and colleagues. Ajit understood at an uncharacteristically early stage of his career that, for a scientist, failures are far more frequent than “eureka” moments of success. In his own words: “if you want something in science—or in life—you must put your heart into it.” We surely know that is how Ajit lived his life and, in doing so, setting an example for all of us. Thanks and may peace be with you, Ajit, wherever you are.
